# Trichothecenes in Food and Feed, Relevance to Human and Animal Health and Methods of Detection: A Systematic Review

**DOI:** 10.3390/molecules26020454

**Published:** 2021-01-16

**Authors:** Magdalena Polak-Śliwińska, Beata Paszczyk

**Affiliations:** Faculty of Food Sciences, University of Warmia and Mazury in Olsztyn, Plac Cieszyński 1, 10-726 Olsztyn, Poland; paszczyk@uwm.edu.pl

**Keywords:** fungi, mycotoxins, trichothecenes, food and feed safety, human and animal health, methods of detection

## Abstract

Trichothecene mycotoxins are sesquiterpenoid compounds primarily produced by fungi in taxonomical genera such as *Fusarium, Myrothecium*, *Stachybotrys*, *Trichothecium*, and others, under specific climatic conditions on a worldwide basis. *Fusarium* mold is a major plant pathogen and produces a number of trichothecene mycotoxins including deoxynivalenol (or vomitoxin), nivalenol, diacetoxyscirpenol, and T-2 toxin, HT-2 toxin. Monogastrics are sensitive to vomitoxin, while poultry and ruminants appear to be less sensitive to some trichothecenes through microbial metabolism of trichothecenes in the gastrointestinal tract. Trichothecene mycotoxins occur worldwide however both total concentrations and the particular mix of toxins present vary with environmental conditions. Proper agricultural practices such as avoiding late harvests, removing overwintered stubble from fields, and avoiding a corn/wheat rotation that favors *Fusarium* growth in residue can reduce trichothecene contamination of grains. Due to the vague nature of toxic effects attributed to low concentrations of trichothecenes, a solid link between low level exposure and a specific trichothecene is difficult to establish. Multiple factors, such as nutrition, management, and environmental conditions impact animal health and need to be evaluated with the knowledge of the mycotoxin and concentrations known to cause adverse health effects. Future research evaluating the impact of low-level exposure on livestock may clarify the potential impact on immunity. Trichothecenes are rapidly excreted from animals, and residues in edible tissues, milk, or eggs are likely negligible. In chronic exposures to trichothecenes, once the contaminated feed is removed and exposure stopped, animals generally have an excellent prognosis for recovery. This review shows the occurrence of trichothecenes in food and feed in 2011–2020 and their toxic effects and provides a summary of the discussions on the potential public health concerns specifically related to trichothecenes residues in foods associated with the exposure of farm animals to mycotoxin-contaminated feeds and impact to human health. Moreover, the article discusses the methods of their detection.

## 1. Introduction

Population in developing countries especially rural areas are dependent on locally produced foods and generally face problems related to food security and mycotoxin contamination which is reflected to be a major food quality issue [1,2]. Chemical contaminants in food and feed are a potential hazard concerning public health. In this sense, mycotoxins are one of the most prevalent contaminants in the food chain [3]. These secondary metabolites produced in nature by a wide variety of fungi, mainly belong to species of *Aspergillus*, *Penicillium*, *Fusarium*, and *Claviceps* genera, which cause food contamination, resulting in mycotoxicosis in animals and humans. Species of the *Fusarium* genus are the most commonly found in temperate regions becoming responsible for worldwide contamination of cereals and feedstuff with the toxins produced [4,5,6]. In this context, mycotoxin contamination is one of the main concerns of all the necessary steps for securing food [7,8]. The contamination of maize and maize-based foods with mycotoxins has been studied frequently [8,9,10]. The most common classes of mycotoxins are aflatoxins (AF), trichothecenes (TCT), fumonisins (FUM), zearalenone (ZEA), and ochratoxin A (OTA) [11,12,13,14,15]. In particular, trichothecene (TCT) mycotoxin is agriculturally more important worldwide due to the potential health hazards they pose. It is mainly metabolized and eliminated after ingestion, yielding more than 20 metabolites with the hydroxy trichothecenes-2 toxin being the major metabolite [16]. This family of compounds is divided into four groups according to their structure, named types A, B, C, and D [16,17,18,19,20,21]. Trichothecenes are groups of chemically related toxins formed by some filamentous fungal species such as *Fusarium*, *Myrothecium*, and *Stachybotrys*. T-2 toxin (T-2), HT-2 toxin (HT-2), neosolaniol (NEO), diacetoxyscirpenol (DAS), monoacetoxyscirpenol (MAS), verrucarol (VER), scirpentriol (SCP), and their derivatives are reported as representative type A trichothecenes [22]. These toxins are manly produced by strains of *F. sporotrichioides* and *F. poae* [23]. Type B trichothecenes are represented by deoxynivalenol (DON) and its acetyl derivatives: 3-acetyldeoxynivalenol (3-AcDON), 15-acetyldeoxynivalenol (15-AcDON), nivalenol (NIV), and fusarenon X (FUS X). These toxins are mainly produced by strains of *F. culmorum* and *F. graminearum* [24]. In addition, types C and D trichothecenes have different chemical structure and are not produced by *Fusarium* species [25]. Therefore, type A and type B compounds are the most relevant trichothecenes, in terms of natural occurrence and high toxicity [26,27]. Although the most toxic groups of trichothecenes are type A (T-2 and HT-2) compared to type B analogues, e.g., DON, NIV, and FUS-X [28]. In addition to these traditional mycotoxins, recently other fungi metabolites have started to be studied since the existing data indicate the toxicity of these compounds. They are called emerging mycotoxins, these metabolites are also mainly produced by species of *Fusarium*, the most representative toxins within this group are fusaproliferin, beauvericin, enniatins, and moniliformin [29,30,31]. The trichothecenes, produced by *Fusarium*, contaminate animal feed and human food in all stages of production and lead to a large spectrum of adverse effects for animal and human health [30,31,32]. Changes in climate conditions in Europe favor an increased level of contamination by such toxins [33,34,35]. Moreover, trichothecenes are very stable to degradation by environmental factors including light and temperature, during milling and other processes [34,35]. They are non-volatile compounds and can be effectively deactivated under strong acid or alkaline conditions. T-2 toxin has been the most extensively studied trichothecene mycotoxin because of its availability and relatively high toxicity [16]. The trichothecene mycotoxins are nonvolatile, low-molecular-weight (MW 250–550) compounds [35]. This group of mycotoxins is slightly soluble in water, but highly soluble in acetone, ethyl acetate, chloroform, dimethyl sulfoxide (DMSO), ethanol, methanol, and propylene glycol. Purified trichothecenes generally have a low vapor pressure, but they do vaporize when heated in organic solvents [2,35].

In consideration of the numerous publications and review articles describing the variety and the toxicological effects of mycotoxins, the current article aims to update a systematic review on the occurrence of trichothecenes in 2011–2020 and their toxic effects and to provide a summary of the discussions on the potential public health concerns specifically related to mycotoxin residues in foods associated with the exposure of farm animals to mycotoxin-contaminated feeds. Moreover, the article discusses the methods of their detection.

## 2. Trichothecenes in Food and Feed

The production of TCT by toxigenic species is determined by genetic factors and the environmental conditions of their growth [34,36]. The fungal propagation and production of mycotoxins is enhanced in developing countries around the world due to conditions like temperature (0 to 50 °C, depending on the fungus species) and moisture levels (≥70%), monsoons, unseasonal rains during harvests, and flash floods [36,37,38]. Researchers have noted that crops in tropical and subtropical areas are more susceptible to mycotoxin contamination, including TCT as compared to those in temperate zones due to the presence of high temperatures and humidity in tropical areas, which provide optimal conditions for toxin formation [39,40,41,42]. Generally, TCT contamination of feeds and food is highly variable, both spatially between different continents, regions, climatic zones, and temporally depending on weather and climatic conditions [43,44,45]. It is also variable at a local level due to differences in soil management and soil types, crop variety and agricultural practices, post-harvest storage, and processing [43]. Considering that animals and humans consume mixed diets from multiple origins, the evaluation of co-exposure to different TCT has been identified as a major task needed to refine their risk characterization because mycotoxin exposure is currently considered to be one of the constraints on production, and a major animal health risk, particularly in monogastric species [43,44]. Organ-specific effects, and even more importantly subclinical effects such as the impairment of the intestinal integrity and effects on the intestinal microbiome are of increasing concerns, as they may occur already at low [43,44]. Great strides have been made in the assessment and management of fungal metabolite risks to human and animal health (monitoring programs in Europe to help protect public health from the risks posed by the major mycotoxins found in foods and feeds, including TCT) [43,44,46,47,48,49]. There are also strong indications that climate change will change the spatial and temporal distribution patterns of TCT production. Continued vigilance and research to increase our understanding of climate-related changes is needed [44]. Consumer safety is the driving-force behind food analysis, as tool used to detect many different classes of residual contaminants in food such as TCT [50]. Toxin production is at the pre-harvest stage and the producing fungal species are also plant pathogens (for example head blight in wheat and other small grains, associated with *F. graminearum* invasion of plants) [51,52]. Toxin concentrations can be measured in the entire plant, but highest concentrations are found in the kernels and particularly the outer hulls [52]. The environmental fate of TCT may be affected by the coexisting presence of bacteria and fungi that can alter their chemical structure and detoxify them [34,36,53,54,55,56,57]. Trichothecenes include a large family of structurally related toxins (closely related chemical compounds called sesquiterpenoids) [2]. Among trichothecenes, type A and type B are of special interest due to their widespread presence and extremely toxic nature.

### 2.1. Type A Trichothecenes 

T-2 and HT-2 belong to the type A trichothecenes with an esterified C-8 moiety. These toxins are produced by the main fungal species such as *Fusarium langsethiae*, *Fusarium poae*, *Fusarium equiseti*, *Fusarium sporotrichioides*, and *Fusarium acumninatum* [1,3,5]. Generally, T-2 and HT-2 toxins can find in barley, wheat, maize, oat, rice, cereal-based food, as well as soybean [16,18,58]. Small grains, such as rice and wheat, are usually contaminated with deoxynivalenol, ochratoxin A, and zearalenone [14,15]. Common analytical methods measure either both individual toxins or the report of sum of both toxins, because during extraction of naturally contaminated materials an interconversion cannot not be entirely excluded [59]. Moreover, plant, microbial, and animal metabolism results in a number of Phase I metabolites, such as 19-OH-T2, neosolaniol and 19-OH-HT2, T2-triol, and T2-tetraol. Relevant conjugates include T2-3-glucose, T2-3-diglucose, T2-3-sulfate, 3-acetyl-T2, 3-feruolyl-T2, HT2-3-glucose, HT2-malonylglucose, HT2-diglucose, as well as the mammalian metabolites HT2-glucuronic acid and T2-3-glucuronic acid [59,60]. Comparison with a large number of analytical data from the EU Member States, from food analyses and from feed analysis, show a large amount of results below the limits of quantitation, and the calculated exposure levels were generally below the health-based guidance levels for humans and animals [59,60]. T-2 toxins are rapidly metabolized within animals and excreted, and no public health concerns are associated with residues in edible tissues, milk, and eggs [16].

### 2.2. Type B Trichothecenes 

DON is a plant pathogenic mycotoxin, and occurs frequently together with its acetylderivatives, 3-AcDON and 15-AcDON [51]. Toxin production by *Fusarium* species (predominantly *F. graminearum*) occurs in small grains prior to harvest and DON and its derivatives can be identified in the entire plant, with often high concentration in wheat straw used for animal feed and bedding [59,60]. In grain ears, the highest toxin concentrations are found in the outer hull and cleaning, sorting, sieving, and dehulling of grains lead to a marked decrease, while increasing the concentrations in cereal by-products such as brans used in animal feeds. Mechanistic studies indicate that DON binds to ribosomes, leading to inhibition of protein synthesis and subsequently also RNA and DNA synthesis. The effect of the binding to ribosomes induces cellular ribotoxic stress and activates different mitogen-activated protein kinases (MAPKs) [59,60]. Activation of MAPKs explains several effects of DON, such as inflammatory effects (release of pro-inflammatory cytokines), apoptosis, and oxidative stress [60]. Moreover, immune toxicity and adverse effects of DON and its derivatives on intestinal integrity are considered to be common adverse effects in animals, probably related to oxidative cellular stress. In contrast to T-2 Toxin group, no haemato- and myelotoxicity have been described [59]. In vitro experiments describe genotoxic effects of DON, which are most likely also related to oxidative stress, as no direct interactions with DNA have been identified [60]. Exposure to DON in foods and feeds was re-evaluated when it became apparent that plant and fungal metabolism results in the occurrence of a variety of modified forms, including relevant concentrations of the DON-3 glucoside (3-DON-Glu) [60]. Upon ingestion, the glucoside bond is cleaved by bacterial metabolism in the intestines, and the freed toxin can be absorbed or transformed into the non-toxic de-epoxidized de-epoxy deoxynivalenol. Hence, it was concluded that risk assessment needs to address all forms of DON, including 3-DON-Glu [42,59,60]. In 2010, the Joint FAO/WHO Expert Committee on Food Additives (JECFA) had already established a group provisional tolerable daily intake (PTDI) of 1 µg/kg body weight per day and a group ARfD of 8 µg/kg bodyweight based on studies in small rodents, indicating a reduced feed intake and body weight gain [59,60]. Even after considering additional human data from incidental intoxications in Asian countries described in the literature, EFSA derived the same PTDI of 1 µg/kg bodyweight per day for infants, toddlers, and other children, and applied this also in the assessment of overall human exposure [42,59,60]. Analysis of food samples in EU Member States revealed exposure rates that are close to the TDI in many cases [59,60]. This correlated with measurements of human urine samples, where the glucuronides of DON and its acetylated forms can be measured with a very high prevalence, confirming regular human exposure via cereals and bakery products [61]. Animal studies revealed that despite a rapid absorption and wide distribution of DON, tissue concentrations are low, and it seems unlikely that residues in animal products contribute significantly to human exposure. Like DON, exposure to nivalenol also does not form residues in edible tissues and nivalenol is unlikely to contribute to human exposure [51,62,63,64]. However, the negative impact of DON and nivalenol on animal health and wellbeing remains of veterinary clinical relevance, particularly in pig husbandry. Lee et al. [65] conducted the study on the detection of major type B trichothecenes, DON, NIV, and their 3-β-D-glucoside conjugates, in food products marketed in Korea and the evaluation of the exposure of the Korean population to these toxins [65]. Researchers analyzed DON, NIV, and their glucosides in 506 foods, which were categorized into six groups using a validated high-performance liquid chromatography (HPLC) method. In these foods, DON, 3-DON-Glu, NIV, and 3-NIV-Glu were detected at rates of 13% (101.9 µg/kg), 8% (22.9 µg/kg), 12% (77.1 µg/kg), and 5% (33.4 µg/kg), respectively [65]. The glucoside conjugate with free toxin was found to occur at the maximum rate, at 49%. The TDI% values of DON, 3-DON-Glu, NIV, and 3-NIV-Glu through food intake in four different scenarios were 1.9–10.2%, 0.4–8.9%, 1.8–23.5%, and 0.5–23.8%, respectively. These results indicate that the estimated exposure of the Korean population to type B trichothecenes is not hazardous. However, continuous monitoring and risk assessment of DON, NIV, and their glucoside conjugates are imperative [65].

Furthermore, for more accurate estimation, the risk of exposure to glucoside conjugates and clear evidence of hydrolysis of these toxins in the mammalian intestine are required, and toxicity studies of co-exposure to glucosides and free toxins are needed [51,64,65,66,67,68]. 

Mwihia et al. (2020) determined the presence, levels, and co-occurrence of mycotoxins in fish feeds in Kenya [69]. Seventy-eight fish feeds and ingredients were sampled from fish farms and fish feed manufacturing plants and analyzed for 40 mycotoxins using high-performance liquid chromatography-high resolution mass spectrometry. Twenty-nine (73%) mycotoxins were identified with 76 (97%) samples testing positive for mycotoxins presence. Mycotoxins with the highest prevalence were enniatin B (91%), deoxynivalenol (76%), and fumonisin B1 (54%) while those with the highest maximum levels were sterigmatocystin (<30.5–3517.1 µg/kg); moniliformin (<218.9–2583.4 µg/kg) and ergotamine (<29.3–1895.6 µg/kg). Mwihia et al. [69] concluded that fish exposure to these levels of mycotoxins over a long period of time may lead to adverse health effects due to their possible synergistic, additive, and antagonist toxic effects. Measures to reduce fish feed mycotoxin contamination should be taken to avoid mycotoxicosis in fish and subsequently in humans and animals through residues [69]. The structurally related trichothecene mycotoxins, commonly found in cereals and derived products are presented in Table 1.

Among trichothecenes, DON turns out the most widespread toxin with the highest level of contamination (Table 1). A different level of DON contamination was found between years in connection with a different meteorological trend, which also varied from year to year. The other trichothecenes (3-Ac-DON, 15-Ac-DON, NIV) were found sporadically, generally in samples showing a high DON contamination [69,70]. In 2020 year, a survey on 120 Algierian cereal samples (barley, maize, rice, and wheat grains) has been carried out to evaluate the presence of 15 mycotoxins (ochratoxin A, deoxynivalenol, fumonisin B1 and B2, T-2 and HT-2 toxins, zearalenone, fusarenon X, citrinin, sterigmatocystin, enniatins A, A1, B and B1, and beauvericin) by Mahdjoubi et al. [70]. With this purpose, a QuEChERS-based extraction and ultra-high performance liquid chromatography coupled to tandem mass spectrometry (UHPLC-MS/MS) were used. Analytical results showed that 78 cereal samples (65%) were contaminated with at least one toxin, while 50% were contaminated with three to nine mycotoxins. T-2 toxin, citrinin, beauvericin, and deoxynivalenol were the most commonly found mycotoxins (frequency of 50%, 41.6%, 40.8%, and 33.3%, respectively). Deoxynivalenol and zearalenone registered high concentrations (48–2055 µg/kg and 10.4–579 µg/kg, respectively) [70]. Furthermore, concentrations higher than those allowed by the European Union (EU) were observed in 21, 8, and 1 sample for fumonisins, zearalenone, and deoxynivalenol, respectively. These results pointed out the necessity of a consistent control and the definition of maximum allowed levels for mycotoxins in Algerian foodstuffs [70]. In addition, the results have pointed out the high co-occurrence of mycotoxins, as well as the high concentration (above the maximum allowed concentration) of some mycotoxins legislated in the EU in those cereals, posing a risk for consumers. These results highlight the necessity of establishing maximum levels for mycotoxin [70]. As regards type A trichothecenes, in presented studies (Table 1) T-2 and HT-2 toxins mainly were detected; however, the EC recommended level of 100 µg/kg was never exceeded. The contamination was quite homogeneous throughout the considered area. Unlike DON, no influence of the preceding crop was observed on T-2 and HT-2 contamination [80]. During both 2017 and 2018, it was noticed that the contamination level of the samples collected in a restricted area (in countries outside of Europe, e.g., Brazil, China, Argentina) was much higher with respect to the other samples [80]. In this same period, the DON contamination in Europe was lower but thanks of several finished studies it was noticed that the average DON content in the analyzed wheat samples exceeded 400 µg/kg. Globalized feed grain trade may distribute TCT outside of their natural occurrence geographical areas, complicating the prediction of TCT mycotoxin contamination in compound feed [80].

## 3. Trichothecenes: Human and Animal Health

The TCT mycotoxins are toxic to humans, other mammals, birds, fish, plants, fand eukaryotic cells, in general [94,95]. The acute toxicity of the trichothecenes varies somewhat with the particular toxin and animal species studied. TCT is hazardously intoxicating due to their additional potential to be topically absorbed, and their metabolites affect the gastrointestinal tract, skin, kidney, liver, and immune and hematopoietic progenitor cellular systems. Sensitivity to this type of toxin varying from dairy cattle to pigs, with the most sensitive endpoints being neural, immunological, hematological, and reproductive effects [17]. The mechanism of action mainly consists of the inhibition of protein synthesis and oxidative damage to cells followed by the disruption of nucleic acid synthesis and ensuing apoptosis. The trichothecenes are a group of structurally related mycotoxins with varying degrees of cytotoxic potency. They have a sesquiterpenoid ring structure and can be classified according to the presence or absence of characteristic functional groups [16]. All trichothecenes contain an epoxide at the C 12,13 position, which is responsible for their toxicological activity. TCT are easily absorbed into the gastrointestinal membranes and are rapidly distributed to various organs and tissues of the body because of their low molecular weight and amphipathic nature [28]. Trichothecenes have a spectrum of adverse effects including emesis, anorexia, growth retardation, neuroendocrine changes, immunotoxicity [20,94,95] and a reduction in food consumption in various animal species (mink, mice, and pigs). The modulation of emesis and anorexia can result from a direct action of trichothecenes in the brain or from an indirect action in the gastrointestinal tract. The direct action of trichothecenes involved specific brain areas such as nucleate *tractus solitarius* in the brainstem and the arcuate nuclei in the hypothalamus [32]. Activation of these areas in the brain leads to the activation of specific neuronal populations containing anorexigenic factors (POMC and CART). The indirect action of trichothecenes in the gastrointestinal tract involved, by enteroendocrine cells, the secretion of several gut hormones such as cholecystokinin (CCK) and peptide YY (PYY) but also glucagon-like peptide 1 (GLP-1), gastric inhibitory peptide (GIP), and 5-hydroxytryptamine (5-HT), which transmitted signals to the brain via the gut–brain axis [32]. Differences are noted among the various species in their susceptibility to trichothecene mycotoxins, but they are small compared with the divergence obtained by diverse routes of administration of the toxins. Once the trichothecene mycotoxins enter the systemic circulation, regardless of the route of exposure, they affect rapidly proliferating tissues [2,30,31,32]. Since trichothecenes induce emesis and growth retardation, mycotoxin contamination is becoming a major issue for child and young animal health [96,97]. The trichothecene mycotoxins are readily absorbed by various modes, including the topical, oral, and inhalational routes [98]. Although the toxicological signature of individual trichothecenes varies to some extent, common features include the inhibition of macromolecule synthesis, particularly protein synthesis, induction of apoptosis and/or necrosis that also affects bone marrow tissue (after T-2 exposure), immunosuppression, and specific organ lesions that are often related to lipid peroxidation. Recently, intestinal epithelial cells have been recognized as an important target for trichothecenes, which affect the tight junction network and subsequently results in impairment of the intestinal barrier function [99]. Loss of barrier function is also followed by an impairment of nutrient transport, the immune system, the increased probability of allergies in humans, and an increased risk from the transfer of pathogens and antigens from the intestinal lumen to the surrounding tissues [100,101,102]. The popular representatives of the trichothecenes are T-2/HT-2 (type A TCT) and DON (type B TCT). The most acutely toxic trichothecene in animals is T-2 while, sensitivity varies among animal species especially in dairy cows, it has been related to feed refusal, gastroenteritis, intestinal hemorrhages, and death while in poultry it causes intestinal lesion and weight loss [11,103]. T-2 toxins are agriculturally among the most important mycotoxins that present a potential hazard to health worldwide, due to the global occurrence of mycotoxigenic molds [104,105]. The T-2 mycotoxin is distinctive in that systemic toxicity can result from any route of exposure, i.e., dermal, oral, or respiratory [104,105,106]. Transmission can occur by direct exposure of contaminated objects and surfaces that have not been appropriately decontaminated [39]. Trichothecenes, especially deoxynivalenol (DON), are known to induce vomiting and anorectic responses in several animal models and in humans [97]. In research reports, it is found that DON concentrations of 1–7 mg/kg diet significantly decrease absorption area of villus surface and also alter the permeability of the gastrointestinal tract resulting in both immunosuppressive and immunomodulating effects in poultry [107,108]. DON toxicity has been associated with animal and human gastroenteritis outbreak resulting in typical acute symptoms such as nausea, vomiting, abdominal pain, diarrhea, headache, dizziness, or fever, also called vomitoxin [109]. Several outbreaks of acute DON toxicity in humans have been reported in India, China, and the USA to strengthen the potential risk for humans [110,111]. The balance between anorexigenic and orexigenic signals that modulate food intake behavior is mediated by several factors of the peripheral and the central nervous systems [112,113].

Trichothecenes cause apoptosis and/or necrosis in the lymphoid, hematopoietic, and gastrointestinal systems resulting in leukopenia, vomiting, and diarrhea that can be lethal. In addition, trichothecenes are toxic to the skin and testes. Immune suppression and increased susceptibility to infection may occur, especially in the late phase of the disease. The toxic effects from trichothecenes largely resemble those following radiation exposure due to effects on rapidly dividing cells in the intestine, bone marrow, and testes. General clinical signs include emesis, food refusal and weight loss, dermal effects, and immune suppression with secondary infection [37]. Clinical signs are dependent on the specific trichothecene involved, the dose, species, route of exposure, as well as the nature of the exposure [37]. Spontaneous and experimental exposures may give somewhat different results, as can exposure to field contaminated materials, when compared to purified toxin. Respiratory, skin, and eye protection is required for personnel working with trichothecenes. There are no specific therapies for trichothecene toxicoses. Neither vaccines nor specific antidotes are readily available [37]. Treatment in people and animals is symptomatic and supportive, and the only known prophylactic measure is avoidance of exposure [37,114]. Trichothecenes increase animal susceptibility to reovirus infection (it is a respiratory enteric orphan virus) and induce an inflammatory response [96]. The T-2 toxin pretreatment also markedly exacerbated the reovirus-induced bronchiolitis and alveolitis, both of which were mild in the control mice. In addition, the pretreatment suppressed the reovirus-induced upregulation of IFN-γ production in the lung while enhancing lung production of IL-6 and MCP-1 [97]. Like T-2 toxin, DON can decrease host resistance to reoviruses. However, it must be given at a much higher dose to achieve this effect. Li et al. (2005) showed that the DON-treated mice were still capable of resolving the infection. This is probably because the mucosal IgA production was enhanced in the DON-treated mice [97]. After a single oral exposure to DON, feed refusal appeared very quickly in mice. Previous risk assessments of DON conducted by the Scientific Committee on Food (SCF) in 1999 and by the Joint FAO/WHO Expert Committee on Food Additives (JECFA) in 2001 identified a no-observed-adverse-effect level (NOAEL) of 0.04 mg DON/kg bw from subacute and subchronic toxicity studies [60].

Savard et al. (2015) showed that high DON concentrations decrease host resistance to porcine reproductive and respiratory syndrome virus (PRRSV) and influence the course of PRRSV infection in pigs [114,115]. However, depending on the dose, DON significantly affected the survival of PRRSV-infected cells and PPRSV replication. In addition, the studies on PPRSV show that trichothecenes may hamper vaccine efficacy. The immunotoxicity of trichothecenes has been studied for more than 30 years. These studies show that trichothecene has complex effects on the immune system [96]. In particular, while trichothecenes are generally immunosuppressive and thereby decrease the ability of the host to control pathogen infections, they can also be immunostimulatory and actually promote resistance to infection. These findings are interesting, because they suggest that the immunostimulatory effect of trichothecenes could be used in the animal husbandry industry to decrease the immunotoxicity of these compounds and perhaps even augment the immunity of farm animals to environmental pathogens [96]. These toxic effects vary according to the chemical structure of the toxin (Table 2). Not only the amount of the toxin but also the duration of exposure determines the degree of such adverse effects.

## 4. Detection of Trichothecenes in Food and Feed

For better understanding of the global effect of mycotoxin contamination, accurate, more rapid, and highly sensitive methods are essential for routine identification and detection of these compounds [116,117,118]. The methods of analysis for TCT are well established and can be applied for cereals, food, feed, and biological samples. Accurate quantification of DON, its acetylated forms, DON-3-glucoside, and other TCT is mostly carried out by liquid chromatography coupled with (multistage) mass spectrometry, presently often with a multianalyte approach. However, this methodology has not been formally validated through interlaboratory studies and proficiency tests have shown that considerable analytical variability exists [2]. Direct approaches (requiring standards) and indirect ones (based on the conversion to DON) have been reported for the determination of DON-3-glucoside, for which the direct approach is the preferred method. For DON, performance criteria for methods of analysis and certified reference materials (both reference matrices and reference calibrants) are available. Non-certified calibrants are available for 3-AcDON, 15-AcDON, and DON-3-glucoside. Immunochemical methods for DON provide rapid and economical alternatives to chromatography, but cross-reactivity and matrix effects have not been fully considered. Recent progress in biomarker research has allowed the determination of DON and its metabolites in urine, primarily as DON-glucuronides, by using single or multiple biomarker methods. However, the commercial sources for the standards of DON-glucuronides are scarce and no (certified) reference materials are available for urinary DON biomarkers [60]. The diverse nature of the matrix, target, environment, time requirements, detection levels, and accessibility of appropriate technology are considered to be challenging [2]. Each approach has merits and drawbacks and the method of choice depends on the detection requirements [64,116]. The matrix effect as the combined effect of all sample components other than an analyte of interest on quantification is very important for mycotoxin analysis because mycotoxin are various chemical entities and present in various sample matrices [116]. Thus, investigation and detection of mycotoxin contamination in foods and feeds have been a vital center of international and national activities over the years. For accurate and rapid determination of these mycotoxins in unprocessed cereals and cereal-based products, sensitive, analytical methods are highly relevant to the toxicological implications to animals and humans and highly desirable in order to measure risk of exposure, further to confirm regulatory levels fixed by the United States, European Union, or different international organizations [119,120] Analysis of mycotoxins usually requires toxin extraction from the matrix (wheat, peanut, maize, etc.) with a suitable extraction solvent, a cleanup procedure such as solvent extraction method, solid-phase extraction, liquid–liquid extraction, accelerated solvent extraction, solid-phase matrix dispersion, DLLME, immunoaffinity column, new absorbents for example graphene oxide—GO and multi-walled carbon nanotubes—MWCNTs, QuEChERS extraction method, and dilute and shooting approaches have been reported with the aim of reducing the matrix effects as much as possible by reducing the interference from the extraction step to analyze multi-mycotoxins [119,120], and determination/detection of the toxin by appropriate analytical instrumentation [2]. Various detectors, namely, fluorescence, UV, laser-induced fluorescence (LIF), mass spectrometry (MS), and photomultipliers (PTM) have been used for quantitative determination.

Derivatization using fluorescence labeling reagents, like 1-Anthroylnitrile (1-AN), is an effective method for determination of trichothecenes [121]. Methods like GC–flame ionization detector (FID)/mass spectrophotometer (MS) and enzyme-linked immunosorbent assay (ELISA) have been used for separation, identification, and quantification of trichothecenes [97,122]. ELISA proved to be a cheap and rapid screening method among others [2].

## 5. Chromatographic Methods

There are many kinds of instrumental detection methods for mycotoxins. Thin layer chromatography (TLC) is a qualitative or semi quantitative method with the longest history in the detection of mycotoxins. High-performance liquid chromatography (HPLC) can couple with different detectors. These detectors include ultraviolet (UV) detection, diode array detection (DAD), fluorescence detection (FLD), or mass spectrometric detection [123,124,125]. Gas chromatography can couple with electron capture detection, flame ionization detection (FID), or mass spectrometry (MS) detection [126,127]. These methods afford high accuracy and precision and are used for both quantitative and qualitative analyses. However, they are expensive, require skilled personnel, and longer periods for sophisticated sample preparation [128]. Thus, instrumental methods are now suitable for normal laboratories in many countries. Chromatographic techniques involving UV and FID are principally employed in confirmatory contexts, thus facilitating compliance with regulations [119,120]. Occasionally, such techniques serve as reference methods for validation of immunochemical tests. In the administration of food safety, mass spectrometry (MS) is becoming more and more important and many analytical methods developed worldwide are based on its application [129,130,131,132]. MS has indisputable advantages of high sensitivity, high selectivity, high throughput and accuracy, making multiresidue analysis possible. Quick, easy, cheap, effective, rugged, and safe (QuEChERS) approaches for sample preparation allow analysis of a wide range of matrices and analytes, and further allowing the simultaneous extraction of the amount of mycotoxins. However, QuEChERS approaches reduce analytical sensitivity, and require preconcentration steps. Alternatively, isotope dilution quantification can improve sensitivity in the absence of preconcentration [124]. High resolution MS (HRMS) and tandem MS/MS allow (possibly) identification of unknown compounds by analyzing structural information of the compounds. The use of non-selective extraction protocols followed by mass screening employing HRMS or MS/MS has allowed identification of new masked mycotoxins and new members of known groups. The rapid multi-residue LC-MS/MS methods have been used to evaluate mycotoxins level in food and feed [124,133,134]. Originally, mycotoxin detection relied on a targeted analysis, with mass spec only analyzing a panel of characterized mycotoxins which may be present in the sample. This legacy approach of detection and quantitation of a target suite was both time-consuming in method development and limited in the scope of what answers could be reported [124]. While this is still a dominant approach for mycotoxins and other residues analysis, advancements in technology continue to expand the capabilities of these analytical methods. Nowadays, innovation has advanced mass spec technology from targeted analysis to a non-targeted or screening analysis. This change has begun through two simultaneous changes in technology. First, instrumentation has become more sensitive, meaning lower-abundance mycotoxins can be identified and the library of known toxins expanded. Secondly, a non-targeted analysis overcomes the need to analyze each toxin individually. Scientists can instead use instrumentation specifically designed to screen samples and identify unknow analytes [124]. DON can be converted to deoxynivalenol-3-glucoside (DON-3-Glu) called as masked mycotoxin by plant detoxification [2]. The analysis of mycotoxins is challenging due to the large number of compounds to be detected and the wide physicochemical properties they possess. In addition, typical food commodity and feed matrices are complex in nature and often contaminated with several mycotoxins at low concentrations [116]. Sample preparation approaches for mycotoxin analysis include liquid–liquid extraction, solid–liquid extraction, matrix solid-phase dispersion, QuEChERS, solid-phase extraction (SPE), and immunoaffinity chromatography [2,64,116]. The considerably different polarity and solubility of the mycotoxins, in particular the polar trichothecenes causes the complications in all approaches. Additionally, the sample extracts may still contain large amounts of matrix components that can negatively affect the detection system. Of course, to overcome some of the limitations of existing methods, there is a need to further develop extraction and clean-up methods for the simultaneous determination of several mycotoxins with high recoveries of the polar trichothecenes and minimizing sample matrix effects [2,64,116]. 

## 6. Ion-Mobility Spectrometry

The ion-mobility spectrometry is a technique used to label chemicals that depend on the velocity achieved by the gas-phase ions in the presence of an electrical field. The working of ion-mobility spectrometry (IMS) is similar to that of Fourier Transform Near Infrared (FT-NIR). The advantages of IMS include low detection limit, simple, fast response and cost-effective [2,135].

Righetti et al. (2018) have developed ion-mobility application to detect different mycotoxins such as HT-2, T-2, DON, in cereal grains [135]. The tests demonstrated high reproducibility (relative standard deviation (RSD) < 2%) in various instrument conditions and were not influenced by complex sample matrices, showing RSD < 0.9%. In traditional LC and gas chromatography (GC) mass spectrometry (MS) workflows, several advantages are attributed to IMS: firstly, it reduces the background noises and provides higher sensitivity in terms of detection of mycotoxins. Secondly this provides additional data on mass spectrum and retention time, the so-called collision cross section, so that compounds can be detected with greater confidence in targeted or nontargeted approaches [2].

## 7. Immunochemical Method

Immunoassays based on antibody–antigen reactions are very useful for routine analyses, as these techniques are simple and have been used for rapid mycotoxin detection [136]. Recently, several immunological techniques have been developed, including enzyme linked immunosorbent assays, time-resolved immunochromatographic assays, enzyme-linked aptamer assays, chemiluminescence immunoassays, fluorescence immunoassays, fluorescence resonance energy transfer immunoassays, and metal-enhanced fluorescence assays [137]. Aptamer is an important parameter in these detection techniques. It can bind a variety of peptides, proteins, amino acids, and organic or inorganic molecules, all of which have high affinity and specificity [138]. Jodra et al. (2015) developed an electrochemical magnetoimmunosensor to detect FB1 and FB2 [139]. The sensor was made of magnetic beads and disposable carbon screenprinted electrodes. Liu et al. (2014) constructed an ultrasensitive immunosensor based on mesoporous carbon and trimetallic nanorattles with special Au cores [140]. The lower detection limit of ZEN was 1.7 pg/mL, and the assay was found to exhibit good stability and reproducibility. As a result of the strong selectivity of molecular recognition mechanisms, it is difficult to simultaneously assay different compounds or discover new toxins. Oswald et al. (2013) designed an analytical array that can detect several targets separately in spatially distinct regions [141]. Song et al. (2014) developed an immuno-chromatographic strip test device that simultaneously detect at least 10 different toxins (AFs, DON and analogs thereof, and ZON and analogs thereof) [142]. Wang et al. (2013) reported that they developed a unique spectral address which can simultaneous detect many mycotoxins in peanuts [143]. Those mycotoxins include AFB1, DON, ZON, and T-2. In comparison to chromatographic methods, immunochemical methods afford greater selectivity in terms of monitoring mycotoxin levels which is very important to ensure food safety in developing countries. In addition, due to global changes in climate and the environment, the level of contamination by fungi and their mycotoxins will increase in the future. Risk management requires routine application of efficient control programs such an optimally employing immunoassays [143]. Analytical methods for trichothecene determination in cereals and cereal-based products presented in Table 3.

## 8. Legislation on Trichothecenes in Food and Feed

Food safety is essential to people’s health and people’s livelihood [131]. It is an important current strategy of the governments, both regulation and standardization are important support for implementing this strategic initiative effectively. The presence of mycotoxins in food and feed has been regulated in many countries. National and international food safety authorities recognize that contamination by mycotoxins and especially by trichothecenes represent a major risk affecting animal and human health [33]. Mycotoxin monitoring programs have been established in all Member States of the EU, and large data sets allow a realistic assessment of human and animal exposure and accompanying health risk, as evidenced by recent surveys conducted in Europe and other countries [43,44].

However, exposure to fungi toxins is not restricted to regulated mycotoxins since these compounds can be modified by fungi, plant, or animal metabolism which leads to products with potential toxicity but not considered in the legislation [67]. These products coming from natural metabolizing processes, and also others resulting from food and feed processing, have frequently been called masked, bound, conjugated, or hidden mycotoxins, but these terms have been used inconsistently [59]. Thus, modified mycotoxins were a term recently introduced to describe all types of mycotoxin modifications and was adopted by the European Food Safety Authority in a scientific opinion about modified forms of certain mycotoxins [59,60]. Many modified mycotoxins have been recently discovered however many others are still unknown. The lack of toxicological studies for the modified toxins together with the hydrolysis reactions that these compounds can suffer during the digestion process are a real hazard for public health that is extraordinarily difficult to assess [59,60]. Care had to be taken with interpretation of the dietary intake results, however, because for a limited number of positive samples mean occurrence levels are strongly affected by the limit of detection of the analytical methods used and this can easily contribute to overestimation of calculated total dietary intake [42].

Due to the toxic effects of trichothecenes on humans, tolerable daily intake (TDI), or provisional maximum tolerable daily intake (PMTDI) values have been set by the U.N. Food and Agriculture Organization/World Health Organization Joint Expert Committee on Food Additives (JECFA) and/or the European Food Safety Authority (EFSA) (Table 4 and Table 5) [160,161,162]. A TDI of 0.1 µg/kg body weight/day for T-2 and HT-2 toxins was set by the EFSA [16]. 

However, recent assessments also revisited the current TDI and proposed an acute reference dose (ARfD) of 0.2 µg T-2 and HT-2/kg body weight. Based on adverse gastrointestinal effects identified from the human outbreak data in China and noting that vomiting occurred within 30 min after an eating occasion, the CONTAM Panel calculated a NOAEL of 26 μg DON/kg body weight for a single eating occasion [60]. By applying a default uncertainty factor of 3.16 for toxicodynamic kinetic differences in the intra-human population variability, a group ARfD of 8 μg/kg body weight per eating occasion was determined. The CONTAM Panel concluded that the dose range calculated from the human biomarker data supported this reference dose [60]. The CONTAM Panel combined the data from both sexes to calculate a BMDL_05_ of 0.11 mg/kg body weight per day for reduced body weight gain, and established a group tolerable daily intake (TDI) of 1 μg/kg body weight per day using the default uncertainty factor of 100 for inter- and intraspecies variability for the sum of DON, 3-AcDON, 15-AcDON, and DON-3-Glu [60]. International Agency for Research on Cancer (IARC) has categorized mycotoxins as proven (Group 1), probably (Group 2A), and possibly (Group 2B) human carcinogen [2]. The United States, the European Commission, and many other countries have established a tolerable daily intake and maximum residue levels (MRLs) for AFB1, OTA, FBs, ZEA, DON (are not recognized as human carcinogens—Group 3), and trichothecene (T-2, HT-2) toxins in different foodstuffs. Due to the common occurrence of regulated mycotoxins (AFs, ZEA, DON, FBs, OTA), their toxic nature has posed a risk to human and animal health, therefore demanding a solution for the protection of fauna [59,60]. Although some toxins have not been regulated such as alternaria, sporidesmins, endophyte mycotoxins, sterigmatocystin, and phomposins, their toxigenic potential has been assessed in various studies (Table 4) [16,59,60]. The toxicodynamic properties of trichothecenes include inhibition of protein synthesis and immunomodulatory effects. Very little information is available relating to their toxicokinetics and toxicodynamics in humans. While there is general agreement that the diet represents an important source of human exposure to trichothecenes, risk assessment from non-dietary routes of exposure is complicated by the limited epidemiological data that are currently available [36] as during the COVID-19 pandemic.

## 9. Conclusions

In recent years, with the fast advancement of detection technologies and introduction of biotechnology, the detection technology of mycotoxins in food and feed, and biological materials, trichothecenes have grown rapidly [167,168,169,170,171,172,173,174,175]. This review summarizes the directions of development of detection technologies of TCT, quantitative methods, and also on the current research of noninvasive detection methods. Sample pretreatment has continuously been a challenging step for analysis of all mycotoxins in various food matrices. Significant advances were made with the introduction of modern IAC and SPE in the cleanup process of samples. Novel nanomaterials have been introduced as absorbents. Different analytical approaches for trichothecenes occurring in cereals and cereal-based products have been advanced and progressively enhanced. Chromatographic methods such as TLC and HPLC are noted to be regular [2]. The developments have passed from the detection of the single compound determination to the concurrent detection of numerous targets, carried out using immense composite cleanup stages, for example, QuEChERS. Among the ordinary methods, immunoaffinity column cleanup linked with HPLC has been the utmost regularly utilized method for the analysis of major trichothecenes in food and feed. LC-MS has all the benefits over HPLC for trace level detection and confirmation, particularly for complex matrices, and it can obtain data regarding this mycotoxin identity. Improvement in analytical chemistry and recent advances in immunochemistry have led to more specific, sensitive, simple, and rapid immunoassays that deliver quantitative and semiquantitative results on-site and have developed as the process of selection for routine analysis of mycotoxins in the field and storehouses [2]. However, the quest for label-free, fast, and more sensitive tools based on immune-biosensor format continues which can offer compact, lightweight, responsive, and reliable mycotoxin detection devices in the field. No doubt, the researchers have described in many publications the pathway of new science, unique technology, inventive material, and state-of-the-art sensing technique but nowadays are not in reach in real life. Although recommending a single method applicable for all types of samples is not possible, the selection of method should be based on the type of sample, the objective, and the facility available in the laboratory. This review will be beneficial for the industries and researchers involved in TCT mycotoxin research to choose appropriate detection and quantification technique.

## Figures and Tables

**Table 1:** Studies on the occurrence of trichothecenes (TCT) distribution (µg/kg) in food and feed samples around the world during 2011–2020.

Food/Feed Matrix	Country	TCT	N Samples	% Positive Samples	Mean [µg/kg]	Range [µg/kg]	Detection Technique	Reference
Barley, maize, rice and wheat grains	Algeria	DON	120	33.3	588	48–2055	UHPLC-MS/MS	[70]
		HT-2		23	18.1	8.4–36.7		
		T-2		100	24.9	16.6–47.2		
Wheat	Poland	DON	92	83	140	10–1265	HPLC	[71]
Wheat	China	DON	181	82	500	33–3030	HPLC	[72]
Wheat	Brazil	DON	172	77	234	73–2794	HPLC	[73]
Wheat	Brazil	DON	48	100	2398	1329–3937	HPLC	[74]
Wheat	Brazil	DON	53	47	641	243–2281	HPLC	[75]
Wheat	Brazil	DON	58	91	360	NA–1310	HPLC	[76]
Wheat	Brazil	DON	745	86	1690	NA–8501	HPLC	[77]
Wheat	Romania	DON	31	26	748	110–1787	LC-MS/MS	[78]
Flour	Romania	DON	35	3	190	NA–190	LC-MS/MS	[78]
Rice	Pakistan	DON	180	8	6.99	<LOD	LC-MS/MS	[79]
Wheat	Italy	DON	293	97	NA	56–27088	GC-MS	[80]
Wheat	Italy	T-2	47	70.2	2.3	0.7–13	LC-MS/MS	[80]
		HT-2	47	85.1	5.7	3–32		
Wheat	China	DON	348	91	240	240–1129	LC-MS/MS	[81]
Wheat	Finland	DON	30	NA	866	NA–5510	LC-MS/MS	[82]
Wheat	Hungary	DON	29	72	NA	NA–1880	ELISA	[83]
Wheat	Argentina	DON	84	100	1750	NA–9480	LC-MS/MS	[84]
Wheat	Switzeland	DON	686	80	607	NA–10600	LC-MS/MS	[85]
Maize	Hungary	DON	29	86	1872	225–2963	ELISA	[83]
		T-2	29	55	69	NA–146		
Oat	Finland	DON	31	100	NA	NA–23800	LC-MS/MS	[82]
Oat	Finland	DON	1672	79	NA	NA–21608	GC-MS	[86]
Oat	Poland	DON	147	31	NA	NA–2975	HPLC-HRMS	[87]
Oat	UK	DON	303	32	28	NA–1866	LC-MS/MS	[88]
Barley	Brazil	DON	76	94	310–15500	1700–7500	LC-MS/MS	[89]
Infant foods	India	DON	29	66	NA	5–228	ELISA	[90]
Corn flour	Serbia	DON	56	42.90	101	NA–931	HPLC	[91]
Pasta and noodles	Germany	DON	40	97	387	60–1609	HPLC	[76]
Cereal-based products	Tunisia	HT-2	32	3	1	<LOD-209	LC-MS/MS	[92]
Bread, wheat grains, wheat flour	Lebanon	HT-2/T-2	137	0	--	--	LC-MS/MS	[92]
Beer, cereal-based food	Spain	HT-2	479	NA	0.047–0.214	NA	GC-ECD	[93]
		T-2	479	0.006	0.215–0.072	NA		

NA—not available in the publication.

**Table 2:** Regulated trichothecenes and their toxicity [2].

Regulated Mycotoxins	Source	Toxicity	References
Type A trichothecenes (T-2 and HT-2 toxin, DAS, NEO)	*Fusarium sporotrichioides, F. graminearum*, *F. moniliforme*, *F. myrothecium*, *F. acuminatum*, *F. culmorum, F. equiseti, Cephalosporium* sp., *Trichoderma* sp.	Immunodepressants, gastrointestinal, mutagenic induction of apoptosis in haemopoietic progenitor cells, effect on protein synthesis, abnormal changes to immunoglobulins	[94]
Type B trichothecenes (NIV, DON, 3-AcDON, 15-AcDON, FUS X)	*Fusarium graminearum*, *F. culmorum*, *F. sporotrichioides*, *F. cerealis, F. lunulosporum*	Immunodepressants, neurotoxic, mutagenic, gastrointestinal.	[56,57]

**Table 3:** Analytical methods for trichothecene determination in cereals and cereal-based products.

Toxin	Matrix	Analytical Method	Detection	Limit of Detection	Reference
TCT	Wheat and maize grains	LC	MS^2^	0.2–3.30 µg/kg	[144]
DON	Wheat and maize	Immunochromatographic strip		50 ng/mL	[145]
DON	Wheat	Direct binding	Electrochemical	6.25 ng/mL	[146]
NEO	Corn	LC	MS^2^	0.90 µg/kg	[130]
T-2				0.40 µg/kg	
T-2 triol				0.20 µg/kg	
DAS				0.65 µg/kg	
3-AcDON				0.75 µg/kg	
15-AcDON				1.60 µg/kg	
NIV				3.00 µg/kg	
FUS X				1.55 µg/kg	
DON, FUS X, 3-AcDON, 15-AcDON, NIV, T-2, HT-2	Cereals, food, feed	GC	MS	2–12 µg/kg	[147]
DON, FUS X, 3-AcDON, NIV, T-2	Wheat, corn	GC	MS	5–10 µg/kg	[148]
DON	Cereals, cereal products	HPLC	UV	30 µg/kg	[149]
DON	Baby food, animal feed	HPLC	UV	6 µg/kg baby food 20 µg/kg feed	[150]
T-2, HT-2, NEO, DAS, DON, NIV, 3-AcDON, 15-AcDON	Maize	HPLC	FLD	10–50 µg/kg	[151]
DON, NIV	Wheat,	UPLC	DAD	20–30 µg/kg	[152]
T-2, HT-2	Wheat, maize, barley	HPLC	FLD	5 µg/kg T-2 3 µg/kg HT-2	[153]
T-2, HT-2	Oats, infant food, muesli, corn grits, breakfast cereals	HPLC	FLD	3 µg/kg	[154]
T-2, HT-2, DAS, MAS, NEO	Oats	LC	APCI-MS	50–85 µg/kg	[155]
NIV, DON, FUS X, 3-AcDON	Maize	LC	ESI-MS^2^	1.5–10 µg/kg	[156]
NIV, DON, FUS X, 3-AcDON, 15-AcDON, DAS, T-2, HT-2	Maize	LC	APCI-MS^2^	0.30–3.80 µg/kg	[157]
NIV, DON, HT-2, T-2, FUS X, 3-AcDON, 15-AcDON, DAS	wheat flour	LC	ESI-MS^2^	10 µg/kg	[158]
HT-2, T-2	Barley	LC	HRMS	5 µg/kg	[159]
DON, 3-AcDON, 15-AcDON,	maize, wheat, barley	HPLC	FLD	3 µg/kg	[133]
DON, T-2, HT-2, FUS-X	Barley, wheat, maize, rice	UHPLC	MS/MS	1–7 µg/kg	[70]

TCT, trichothecenes; DAS, diacetoxyscirpenol; DON, deoxynivalenol; 3-AcDON, 3-acetyl-deoxynivalenol; 15-AcDON, 15-acetyl, deoxynivalenol; FUS X, Fusarenon X; MAS, Monoacetoxyscirpenol; NEO, neosolaniol; NIV, nivalenol; T-2, T-2 toxin; HT-2, HT-2 toxin; GC, gas chromatography; LC, liquid chromatography; MS, mass spectrometry; MS^2^, tandem mass spectrometry; ESI, electrospray ionization; APCI, atmospheric-pressure chemical ionization.

**Table 4:** Recommended maximum tolerable daily intake for major TCT mycotoxins.

Mycotoxin	TDI or PMTDI	Type	Basis	References
Deoxynivalenol and acetylated derivatives	1.0 µg/kg body weight/day	PMTDI	Rodent nephrotoxicity	[160]
Nivalenol	1.2 µg/kg body weight/day	TDI	White blood cell reduction-rats	[161]
T-2 + HT-2 toxins	0.06 µg/kg body weight/day	PMTDI	White and red blood cell reduction-swine	[162]

TDI—tolerable daily intake; PMTDI—provisional maximum tolerable daily intake [52,163,164].

**Table 5:** Maximum levels for mycotoxins in cereals and cereal products for human consumption [165,166] and in products intended for animal feed [161,162].

Mycotoxin	Cereal And Cereal Products	Maximum Levels [ug/kg]
**Human Consumption**		
DON	Unprocessed cereals other than durum wheat, oats and maize	1250
	Unprocessed durum wheat and oats	1750
	Unprocessed maize, with the exception of unprocessed maize intended to be processed by wet milling	1750
	Cereals intended for direct human consumption, cereal flour, bran and germ as end product marketed for direct human consumption	750
Sum T-2 and HT-2 toxin (*)	Unprocessed cereals	
	Barley and maize	200
	Oats	1000
	Wheat, rye and other cereals	100
Sum T-2 and HT-2 toxin (*)	Cereals grains for direct human consumption	---
	Oats	200
	Maize	100
	Other cereals	50
**Animal Feed**		
DON	Feed materials	---
	Cereals and cereal products with the exception of maize byproducts	8
	Maize byproducts	12
	Complementary and complete feedstuffs with the exception of: Complementary and complete feedstuffs for pigs Complementary and complete feedstuffs for calves (<4 months), lambs and kids	5
		0.9
		2
Sum T-2 and HT-2 toxin	Cereal products for feed and complementary feed	---
	Oat milling products	2000
	Other cereal products	500

* Indicates recommendations.

## Data Availability

Not applicable.
